# Assessment of Mechanical and Thermal Properties of Hemp-Lime Mortar

**DOI:** 10.3390/ma14040882

**Published:** 2021-02-12

**Authors:** Eliana Parcesepe, Rosa Francesca De Masi, Carmine Lima, Gerardo Maria Mauro, Maria Rosaria Pecce, Giuseppe Maddaloni

**Affiliations:** 1 Department of Engineering, University of Sannio, Piazza Roma, 21, 82100 Benevento, Italy; rfdemasi@unisannio.it (R.F.D.M.); germauro@unisannio.it (G.M.M.); pecce@unisannio.it (M.R.P.); maddaloni@unisannio.it (G.M.); 2 ITEMS SRL, Piazza Guerrazzi, 1, 82100 Benevento, Italy; c.lima@tesis-srl.eu

**Keywords:** ecofriendly materials, natural fibers, hemp-lime mortar, experimental tests, environmental conditioning, aging

## Abstract

The use of renewable and natural materials characterized by the low environmental impact is nowadays a key issue for the sustainable development of the construction industry. For this reason, the interest for natural fibers, to be used as reinforcement in composites as an alternative to other fibers, is continuously growing. In this paper, the use of hemp for reinforcing lime mortar used as plaster is considered with a multidisciplinary approach, taking into consideration the structural and thermal performance. Natural fibers have several advantages compared to industrial ones, such as low cost, low environmental impact, biodegradability, renewable nature. Moreover, these can show remarkable mechanical performance in relation to specific weight, and sometimes, as in the case of hemp fibers, these can improve the thermal insulation capacity of the plaster. However, the experimental results on the mechanical features are still lacking, especially to assess their durability, and the variability of thermal parameters with the mechanical characteristics. Therefore, this paper proposes an experimental program, developed at Laboratory of Materials and Structures (LAMAS) of the University of Sannio (Italy), aimed at investigating the main mechanical properties (compression strength, flexural strength) of lime mortar reinforced by hemp fibers and subjected to various environmental exposures and aging processes. The characterization is completed with the measurement for the produced samples of the thermal conductivity by means of the standardized guarded hot plate technique.

## 1. Introduction

In recent years, environmental sensitivity has led to a renewed interest in sustainable buildings and issues, such as recyclability and environmental safety are becoming increasingly important in the construction sector.

From this perspective, eco-compatible materials are playing an important role in the building materials market, in particular, natural fibers, which are used to reinforce different types of composite materials, such as lime-based binder products [1,2]. Ecofriendly materials should be respectful of the three pillars of sustainable development: environmental, economic, and social.

The use of natural fibers in composite materials presents several advantages compared to others, such as synthetic fibers, including low environmental impact through all their life cycle and low cost that supports their potential use. Indeed, the production of natural fibers is primarily based on solar energy, and it needs a small amount of energy from fossil fuels. Moreover, natural fibers are biodegradable, lightweight, renewable, nonabrasive, abundant, and are an important source of income for the population living in rural areas [3,4,5]. The most appealing advantages of natural fibers are their beneficial impact on the environment [6]. About this topic, recently, Chen et al. observed that the natural fiber-reinforced polymer achieved lower impact according to six indices of the life cycle assessment [7]. For instance, the production process of reed fibers consumes only 7.5% energy, emits 32.3% carbon dioxide, 20.6% biochemical oxygen compared to glass fibers of the same weight [8]. The adoption of kenaf to replace glass fiber as reinforcement in cement panels reduces the environmental impact significantly on human health, natural resource depletion, and habitat alteration [9] due to the production phase of cement (over 60% of total impact). Moreover, most natural fibers, originating from agriculture crops, are abundant, biodegradable, and renewable materials [10].

The proposed research activity has the aim to investigate the main mechanical properties that characterize a plaster, such as flexural and compression strength, in order to manufacture bio-compatible and sustainable plaster, such as hemp-lime mortars. After a review of the state-of-art, the results of an experimental program are presented to understand the effect of various environmental exposures, such as humidity and marine conditions, on the mechanical properties of the composite material in order to analyze the durability issue that could affect its performance. For the most interesting sample, the thermal characterization is also performed for discussing the influence of hemp inclusion on the insulation property. This combined approach opens interesting perspectives for identifying key compositions that need to be optimized for a better selection of hemp fibers’ characteristics.

It must be remarked that a complete characterization of hemp-lime mortar would require numerous laboratory tests because the performance of the composite is related to many factors, such as the morphology of the fillers, the orientation of the filler particles, porosity, the method and the ratio of compaction, the distribution of the fillers, and many others. However, in this paper, the authors propose the tests for two mixtures in terms of structural and thermal behavior to discuss a preliminary approach and characterization for evaluating the possibility of a product that has a double function.

## 2. State-of-Art

Between natural materials, there is a renewed interest in hemp growing because the European Union reintroduced the legal cultivation of industrial hemp with a THC (Tetrahydrocannabinol) content lower than 0.2% [11,12]. Hemp fibers are the most valuable part of the plant, and their main constituents are cellulose, hemicellulose, lignin, and pectin. Diameters and properties vary significantly depending on the source, age, retting and separating techniques, geographic origin, rainfall during growth, and constituents’ content [13]. The hemp species used for fiber production with the industrial application is Cannabis sativa. In more detail, the hemp products are particularly interesting since these do not require agrochemicals in their cultivation and, in common with all similar plants, transform carbon dioxide during their rapid growth and capture carbon, releasing the oxygen into the atmosphere [14]. Campiglia et al. [15], considering the life cycle assessment and the carbon footprint for different hemp genotypes, found that the impacts grew by decreasing both nitrogen fertilizer and plant densities. Briefly, these plants grow prolifically with limited resources, and the low impact is due to their ability to thrive in natural environments. The main advantages [16] consist of the fact that synthetic fertilizers are not needed, the plants require less than a third of the water needed for cotton, and these can be grown in many climates and soil types. These characteristics suggest that the production and processing costs can be lower than those for current components of traditional materials. Moreover, hemp fibers are lightweight, and this allows lower costs for transportation and handling, and thus these can be an interesting alternative in the rural zones where the production waste can also find other uses. The ease of hemp production could promote a significant supply chain planning, reducing distances between producers and potential users, and generating small and medium enterprises engaged in the production of various construction products made of hemp (panels, blocks, plaster, etc.). Therefore, large-scale use of this material could lead to local economic benefits, also supporting the growth of rural communities.

The life cycle assessment of hemp fiber highlights further advantages like high biomass yield, ability to extract heavy metals from the soil, and low eutrophication potential [17]. These aspects are very important considering the growing attention to the sustainability of the building market and the problem of energy prices and shortages of local fossil energy sources. In addition, the energy consumption during the building use needs to be minimized. From this perspective, the insulation property of construction materials is the main characteristic. Thermal insulation produced from more natural raw materials requires less energy than is produced from artificial raw materials. According to [18], the thermal conductivity of hemp insulation is comparable with other insulation materials, but the energy consumption required for producing is three times lower than that needed for extruded polystyrene.

Regarding the mechanical properties of hemp, several authors investigated the tensile behavior of natural fibers, and their investigations pointed out a large discrepancy among values reported for tensile strength and Young’s modulus [19,20]. Indeed, as natural fibers generally present variable and irregular cross-sections, their measurement can lead to huge errors in the computation of stress. Despite scientific evidence that proves that the tensile properties of hemp fibers are good enough to be used as composite reinforcements showing an almost linear stress-strain curve during the whole test [21], the relatively high moisture content of fibers, the variability in the properties of the fibers, and the relatively poor fiber/matrix interfacial strength can limit their use and reduce their mechanical efficiency.

About the application in the building sector, hurds and fibers obtained by the processing of hemp are used in the manufacturing of bio-composites, concretes, and insulation mats [22]. Hemp concrete is generally suitable to form building envelopes by casting between, or spraying against, temporary or permanent shuttering in situ or by pre-fabrication of building blocks or panels [23]. Some papers underline the good thermo-acoustic properties and the transpirability and hygroscopicity of these products that can contribute to improving comfort and indoor air quality [24].

Hemp bio-composites have been investigated by many authors for production from several polymer materials, such as polypropylene and polyethylene, of reinforced materials with high-performance and specific mechanical properties [25,26]. Sassoni et al. [27] presented novel hemp-based composite materials, in the form of panels, produced by bonding hemp hurds with a new hybrid organic-inorganic binder, with a thermal conductivity value of 0.078 W/(m·K) for the low-density panels. Hemp fibers also show good performances as green reinforcement, leading to composites with good physical and mechanical properties if their low price and density are considered as terms of comparison with respect to industrial fibers [4]. However, the variability of the final mechanical properties of composites with natural fibers as reinforcing materials can make it difficult to predict their performance with any degree of precision [28,29].

Hemp-fiber mats seem a suitable alternative for building thermo-acoustic insulation with better results in terms of an environmental impact compared with traditional insulation materials. Most of these are manufactured from either mineral or petro-chemical resources with very energy-intensive manufacturing processes. For instance, Zampori et al. [30] performed a comparison between hemp and a rock-wool mat, concluding that the former was far less impacting than the second. This was attributed to the carbon dioxide emissions associated with hemp-plant photosynthesis. Instead, Kymäläinen and Sjöberg [31] attributed this benefit to the fiber’s biodegradability, which enabled the reduction of the environmental impact at the end-of-life phase. Fassi and Maina [32] underlined that, compared to other natural materials and, most of all, to mineral and synthetic materials, hemp fibers caused slight impacts in terms of consumption of non-renewable resources and energy for production and processing and, also, for disposal at the end-of-life. In contrast, they documented that hemp fibers caused no impacts on ozone-layer depletion, the greenhouse effect, and acidification.

Furthermore, hemp fibers and hurds can be used in composites [33,34] with a mortar matrix, giving good solutions, especially for plasters, that become suitable also for energy saving. Indeed, considering the objective of carbon neutrality by 2050, these materials can contribute to the reduction of building’s operational energy use as well as the adoption of natural, renewable, and low embodied energy building sources in the construction process. The performance of hemp-lime composites seems promising, also in terms of environmental impacts, due to the active carbon sequestration of the hemp plant during its growing phase [35] and the gradual sequestration of carbon emitted during the production of lime. The thermal properties of hemp-lime materials are found comparable to those of commonly-used lightweight insulation material with similar density [36]. However, the available research underlines that the optimization of the mechanical properties of hemp concretes can affect the value of achievable thermal conductivity [37]. For this reason, it is important to carry out both characterizations for new materials with the aim to develop a product that is structurally functional, energy-efficient, and environmentally sustainable.

Despite the ecological and sustainable advantages, the use of natural fibers in composite material has also disadvantages: quality and production efficiency, which depends on the natural conditions, heterogeneity of their properties, which may be associated with the manufacturing process, and the hydrophilic behavior, which leads to water absorption in the composite materials [3]. Furthermore, the mechanical performance as tensile strength, compression strength, and especially impact strength of natural fiber composites are relatively low compared, for example, to glass fiber composites, even if natural fibers can easily compete with glass fibers in terms of stiffness [38].

Hemp composites have been widely used over the last two decades for non-structural components and as fiber-reinforced mortars for masonry blocks in the “heavy” masonry building sector. Hemp mortar or hemp concrete is a bio-composite construction material obtained by mixing generally a lime-based binder that acts as a matrix, hemp shives, and fibers that are obtained by processing of hemp stem and water as a reagent.

As summarized by Brzyski et al. [39], the hemp-lime composites are usually characterized by a low apparent density (256–627 kg/m^3^) with a total porosity in the range of 72–80% [40], low diffusion resistance coefficient (around 5–6) [41], high mass absorptivity (110.8–134.5% for the composites with an apparent density of 411.6–438.7 kg/m^3^) [42].

In the literature, several studies have been carried out on the mechanical and physical characteristics of this type of light composite. The compressive strength is one of the more common tests carried out on hemp-lime composites. For instance, Kremensas et al. [43], by using a dry incorporation method of corn starch and hemp shiv, found compressive stress at 10% of deformation in the range of 2.4–3.0 MPa, bending of 4.4–6.3 MPa, and tensile strength of 0.23–0.45 MPa.

Various research results show that numerous parameters influence the mechanical behavior of hemp composite: the effect of curing conditions, the mixing method, the binder characteristics, and hemp characteristics, such as the fiber length and the fiber content by weight [44,45,46,47]. Li et al. [28] found that the addition of hemp fiber into the concrete matrix resulted in a linear reduction in the specific gravity and the water absorption ratio. The compressive strength, flexural strength, and toughness indices are all correlated with empirical regression equations to aggregate size parameters, fiber factors, and matrix initial mechanical properties.

In general, the results obtained on the lime-hemp concrete indicate that the mechanical resistance of the material depends strongly on its density, and the compressive strength increases with time [48]. Regarding the hemp content, optimal results in terms of strength are obtained for cement mortars reinforced with a 2–3% amount of natural hemp fiber [49,50]; otherwise, a higher percentage of hemp could almost completely inhibit hydration reactions.

Another studied aspect is the adoption of recycled and locally sourced materials for improving the properties of hemp-lime composites. For instance, Abdellatef et al. [51] experimented with the use of recycled crushed brick as a pozzolan to create more environmentally-friendly hemp-lime composites. Their results indicated that mixes with a 1:1 binder to hemp ratio and 300–400 kg/m^3^ density had hygrothermal and mechanical properties suitable for insulating infill wall applications.

Furthermore, a critical issue for composite materials with natural fiber as reinforcement is durability because the physical and mechanical properties of the hemp fibers could be influenced by environmental conditions, such as alkali attacks and water adsorption, which could lead to degradation phenomena [52,53].

The research on the application of flax and hemp is concluded with positive results, also in the case of thermal performance. Indeed, Brzyski et al. [54] found that for the lightest experimented mixtures, the compressive strength was below 1 MPa, and the thermal conductivity below 0.1 W/(m·K), making them better than autoclaved aerated concretes. The study proposed by Curto et al. [55] has shown that the increase in the percentage of hemp shives in a mixture of lime and water decreases the thermal conductivity and increases the sound absorption values. Pochwała et al. [56] found for a hemp-lime composite that the thermal conductivity ranged from 0.038–0.055 W/(m·K), depending on how much the shiv was compacted and what mixing method was applied. These properties are attributable to the porosity between the particles (about 80%) and the appearance of a porous surface [57]. The high porosity, which combines the microscopic pores of its binder and its vegetable particles and bigger pores between the particles, provides not only a low thermal conductivity depending on the mix formulation, water content, density [58] but also a high permeability and, consequently, a good acoustic absorption (higher than 0.8) [57]. Furthermore, these materials exhibit a high hygroscopic behavior [59], which allows energy savings and high indoor comfort [24] as hemp fibers are natural regulators of environmental humidity.

Thanks to their thermal performance, the hemp composites have been positively considered for the building refurbishment or restoration [60]. Some papers discuss the energetic performance of historic buildings after the application of hemp-lime compared with gypsum lime [61]. For instance, Agliata et al. [62], using a case study “Palazzo Jadicicco”, found that the overall energy performance of the building increased by around 20% with a one-level improvement of the building energy class, from E to D. Besides, if it is a good result, it should underline that the decrease in thermal transmittance was not sufficient to fulfill the regulatory requirements, in Italy.

However, it must be remarked that the improvement of thermophysical properties is usually matched with a reduction in the mechanical performance; thus, it is important to analyze the effect of fibers on the strength and elastic response of the composite hemp-lime mortar.

In the following sections, the results of an experimental campaign developed at the LAboratory of MAterials and Structures (LAMAS) at the University of Sannio (Benevento, Italy) framed within the above topics are illustrated, and the effects deriving from the use of hemp components and the environmental conditioning are highlighted. The tests for the thermal conductivity have been carried out in the Insulating Material Thermal Analysis Laboratory (IMATlab) of the University of Naples Federico II (Naples, Italy).

## 3. Experimental Set-Up

### 3.1. Materials

Mortars were prepared using a commercial, natural hydraulic lime with silica sand and dolomitic limestone aggregates (Kerakoll, Sassuolo, Italy), applied as plaster. The natural origin of its ingredients guarantees compliance with the fundamental parameters of porosity, hygroscopicity, and required breathability, making it particularly suitable for construction, renovation, and recovery mortar. According to the European Standards [63,64], the binder belongs to the class designated as ‘‘NHL 3.5” regarding the chemical composition and to the class “M5” regarding the strength (compression strength of 5 MPa).

**Figure 1:**
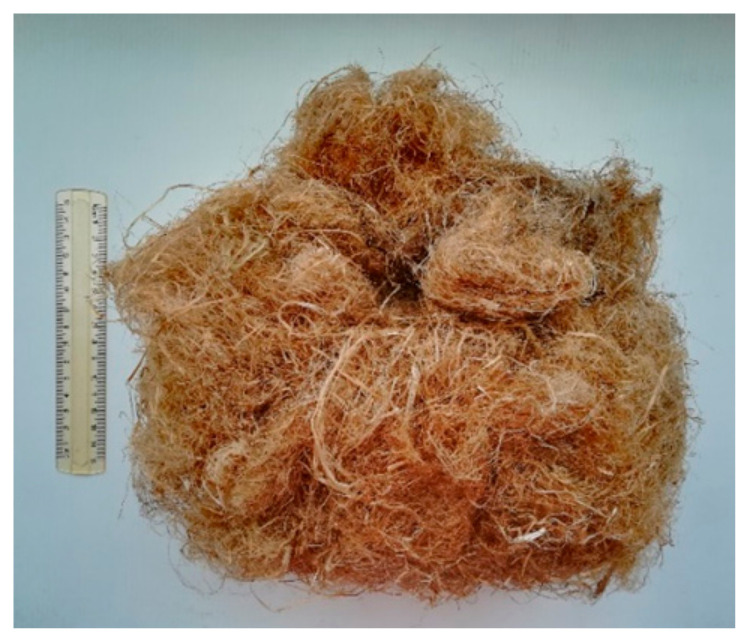
Hemp fibers used in the experimental program.

In this research, untreated hemp fibers (Cannabis sativa) (Campanapa Sca, Benevento, Italy) selected with a diameter in the range 50–200 μm and a density of approximately 1500 kg/m^3^ were used. To manufacture the composite, the hemp fibers were combed, cut to 10–15 mm in length, and then added at 0.2% in weight with respect to the binder (Figure 1). The quantity of hemp fibers used was assessed for workability issues after trying to manufacture mixtures with other fibers percentages.

The dry mixture was initially homogenized manually, and then water was added gradually with a water/lime ratio by weight of 0.18 as indicated in the manufacturer’s technical specifications. Subsequently, the water/lime ratio was updated in order to check the influence of the manual manufacturing procedure so as to simulate the real conditions of installation in situ, which has an effect on the workability and mechanical response of the mixture.

The mixture was casted into prismatic molds measuring 160·mm × 40·mm × 40 mm for mechanical tests and 300 mm × 300 mm × 50 mm for thermal tests for which the thickness was verified by means of digital vernier caliper with 0.1 mm accuracy. After the setting of the plaster, the specimens were demolded. In addition, reference specimens without hemp fibers were also manufactured. Overall, 2 types of the mixture were prepared on different days, considering first the manufacturer’s technical specifications (Mix 1) and then updating the water content (Mix 2) to improve workability and facilitate the manual mixing with hemp fibers: a total of 24 fiber-reinforced samples (FR) and 14 reference samples (REF) without hemp fibers for mechanical tests and 2 FR samples and 2 REF samples for thermal tests (Table 1).

**Figure 2:**
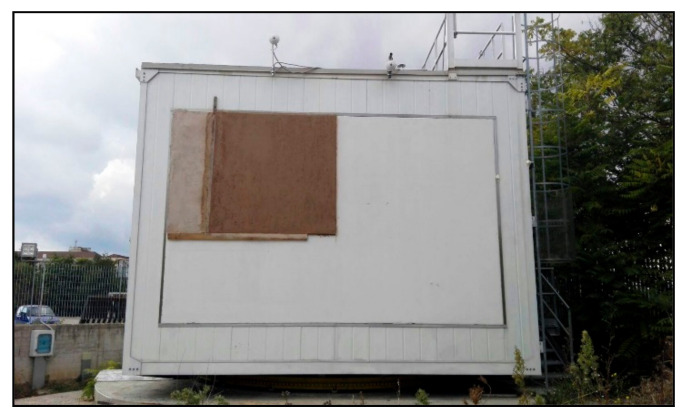
Hemp-lime mortar (mixture 1) applied on Matrix test room.

During the preparation of the specimens, also the workability was examined considering the ease of installation and the homogeneity with a visual inspection of Mix 1 as plaster. The obtained hemp-lime mortar has been applied as the finishing layer of a sector of the wall of MATRIX that is the test room of the Department of Engineering of the University of Sannio [65]. It is a large-scale test room (36 m^2^ floor area), equipped with a great number of sensors for the characterization of the building elements. Figure 2 shows the application of mixture 1 in a section of the test room, which will be interesting in the future months of in situ evaluation of heat fluxes and surface temperature measures.

The in situ inspections demonstrated that the considered mixture assured fast drying and good adhesion on the surface. The mortar clung to vertical surfaces and resisted flow during the placement.

### 3.2. Experimental Methods and Measurements of Mechanical Tests

**Figure 3:**
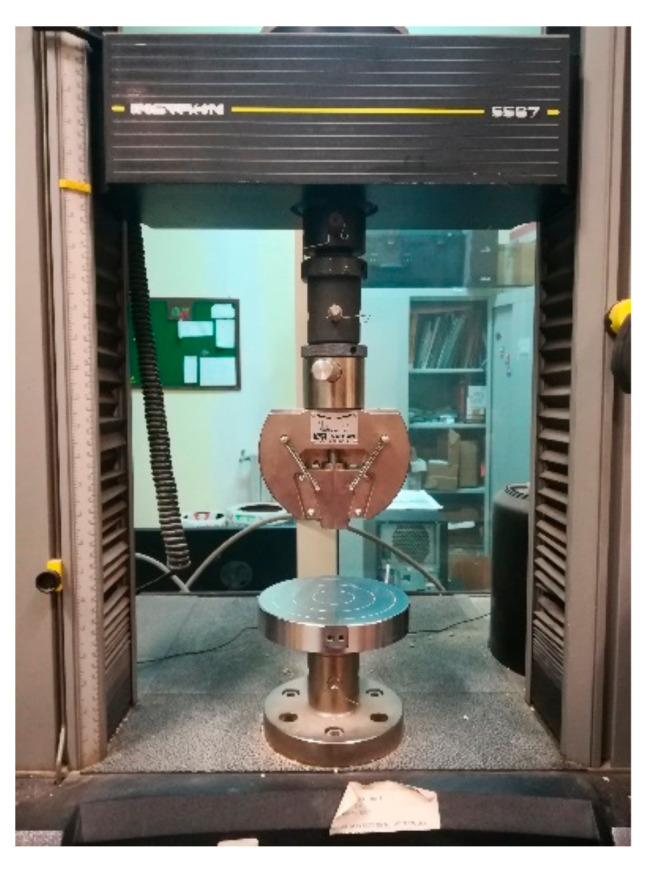
Universal testing machine.

Mechanical characterization of the hemp-lime mortars was carried out according to European Standards [66]. Firstly, three-point bending tests were performed using an Instron 5567 compression-tension machine (Figure 3). At the end of the test, the flexural strength of the specimen f_t_ was calculated using the following Equation (1):

(1) $$f_{t}=1.5\frac{F\times l}{b\times d^{2}}$$

where F is the maximum load supported by the specimen, l = 100 mm is the span length between the supports, b = 40 mm and d = 40 mm are, respectively, the breadth and the depth of the specimen section.

Then, compression tests were carried out on the two fragments of each sample resulting from the above bending test, using the same testing machine. The compression strength of the specimen f_c_ was calculated by the well-known Equation (2):

(2) $$f_{c}=F/A_{p}$$

where F is the maximum load supported by the specimen, and A*_p_* is the area of the auxiliary plates used to apply the load having a size of 40 mm × 40 mm.

Each test was performed in displacement control with a constant speed of 0.5 mm/min.

#### Environmental Conditioning

**Figure 4:**
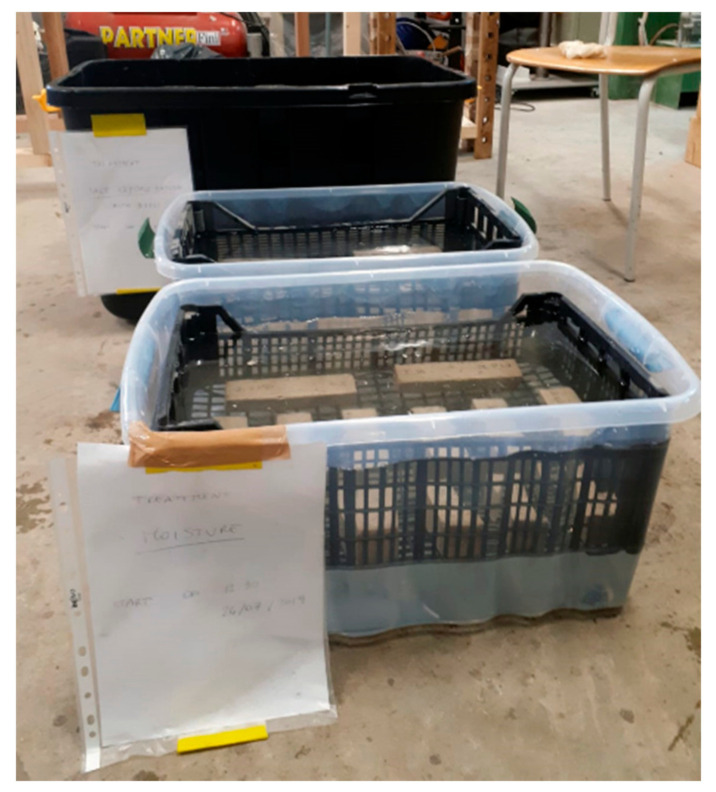
Specimens immersed in exposure environments: the saltwater environment in the black tank, moisture environments in white tanks.

In order to study the durability performance of the material, the specimens were subjected to conditioning by immersion in tanks where exposure environments were reproduced (Figure 4).

In particular, the exposure environments selected for this study, made in accordance with the reference standards, are the following:

1. Saline environment (SE): the environmental conditioning was carried out by immerging specimens in saltwater according to ASTM D1141 for 1000 h (S1000) and 3000 h (S3000) [67].
2. Moisture environment (ME): conditioning was done by immerging specimens in tap water for 1000 h (M1000) and 3000 h (M3000).

Table 2 summarizes the tested specimens according to the type of mixture, the presence of hemp fibers, and the type of conditioning. In particular, it can be noted that:

1. Mix 1 had both conditioned specimens (C) and not-conditioned specimens (NC) in order to compare the results due to the environmental conditioning and also to control the effect of hemp fibers reinforcement under normal conditions and exposure environments.
2. Mix 2 had REF specimens only in normal condition (NC), while FR specimens were both not-conditioned (NC) and conditioned (C) in order to analyze the effect of hemp fibers reinforcement under normal conditions and to study the exposure effects on specimens with hemp fibers.

### 3.3. Experimental Methods and Measurements of Thermal Tests

**Figure 5:**
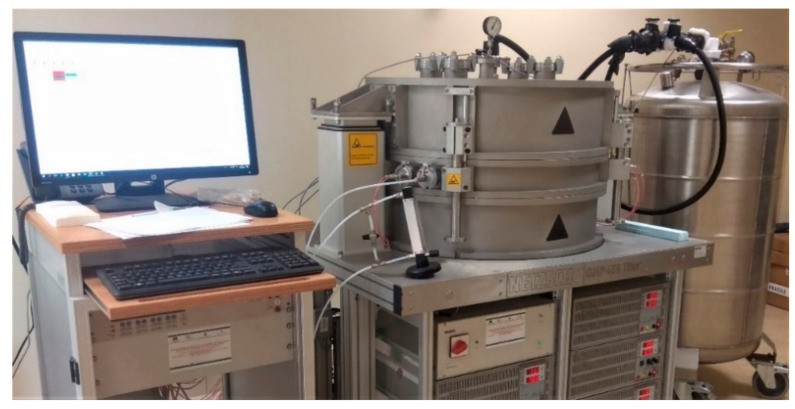
NETZSCH GHP 456 Titan^®^ system.

With regard to the thermal conductivity (λ) measurements, the NETZSCH Guarded Hot Plate (GHP) 456 Titan^®^ system (NETZSCH-Gerätebau GmbH, Wittelsbacherstraße 42, 95100, Selb, Germany), a high-temperature version, was used [68]. Based on the well-known standardized guarded hot plate technique, ISO 8302 [69], the system implemented an absolute measurement method—thereby not requiring calibration standards—and allowed a wide operating temperature (T) range (−160 °C to 600 °C) without the risk of thermally-induced deformation. The GHP 456 Titan^®^ looks like as in Figure 5, consisting of a cylinder over a separate control system and stabilized power supply, ensuring fast attainment of programmed plate temperatures and perfect stability.

**Figure 6:**
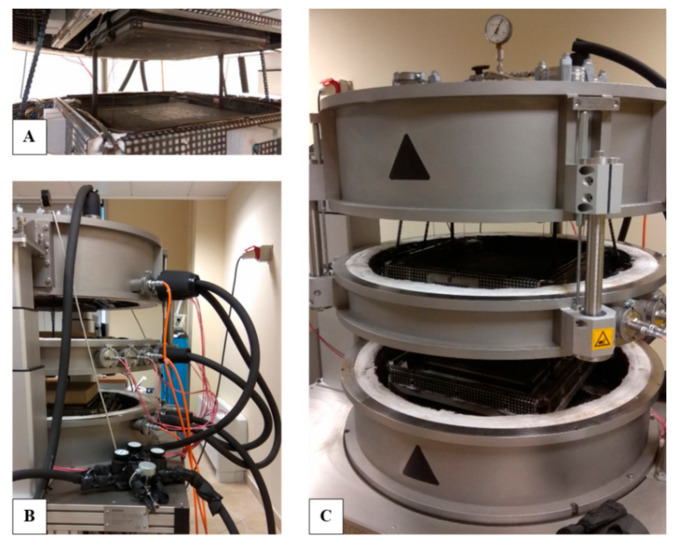
GHP 456 Titan: (**A**) focus on a hot plate and guard ring (below), cold plate (above); (**B**) thermocouples and insulated pipes for N2; (**C**) open-system configuration.

As depicted in Figure 6, control, stabilized electrical supply, and cooling systems—using liquid nitrogen in a connected tank—were employed to achieve the desired temperature levels with high stability and accuracy, around 2%, as shown in Table 3, where the system technical specifications are reported. On the backside, and thus not visible in Figure 6, there were further feedthroughs for temperature sensors, for heaters, and for cooling lines for the cold plates, as well as a flange for evacuating the instrument.

As for the working principle, the hot plate and the guard ring are sandwiched between two samples of the same material with approximately the same thickness (t). The cold plates are placed above and below the samples, respectively. All plate temperatures are controlled through the heating (electrical power supply) and cooling (liquid nitrogen) systems so that a defined temperature difference (ΔT)—set by the user—is established between the hot and the cold plates, i.e., across the sample thickness. The guard ring is kept at the hot plate temperature to minimize lateral heat losses. Once reached steady-state conditions, the material λ value—at the set T and ΔT—is assessed according to the Fourier law Equation (3):

(3) $$\lambda =\frac{\dot{Q}\times t}{\Delta T\times 2\;A}.$$

where:

1. $\dot{Q}$ is the thermal power input to the hot plate, generated electrically, thanks to the Joule effect.
2. t is the average thickness of the sample, and ΔT is the temperature difference, as aforementioned.
3. A is the front area of the sample, generally equal to the hot plate area, i.e., 0.30 m × 0.30 m = 0.09 m^2^; otherwise, filling materials (spacers) have to be used.
4. Factor *2* is present because the measure is performed on two samples.

## 4. Experimental Results

### 4.1. Results of Mechanical Tests for NC Specimens

The mean value of flexural tensile strength f_tm_ (MPa) and the respective coefficient of variation COV_t_ for each type of mixture are determined. For the compression behavior, the mean values and the coefficients of variation for each type of mixture, f_cm_ (MPa) and COV_c_, respectively, are also defined. Detailed results of each specimen are reported in Appendix A.

**Figure 7:**
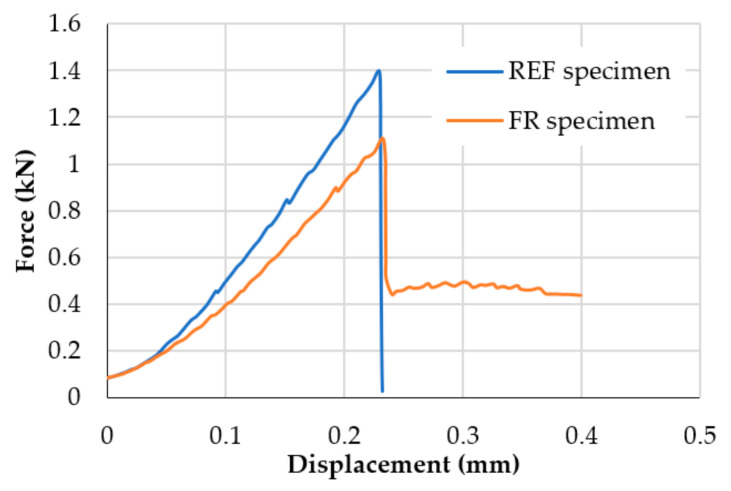
Comparison of experimental curves of bending test: specimen I2 (REF specimen) and specimen IFR2 (FR specimen).

In general, the three-point bending tests have evidenced that the presence of fibers changes the failure mode of mortars from brittle (REF specimens) to ductile (FR specimens), increasing the ultimate displacement (Figure 7).

**Figure 8:**
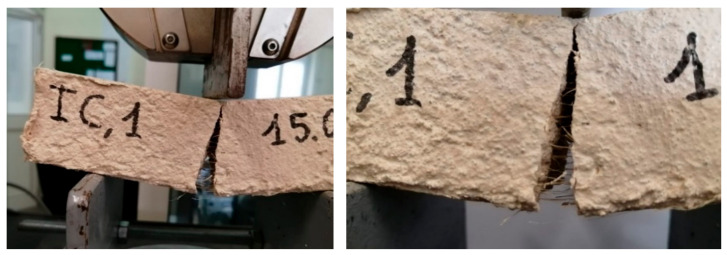
Sewing effect of fibers at the bending test.

This result is due to the stitching effect of the fibers (Figure 8). Indeed, the addition of fibers in the inorganic matrix could improve the toughness of the material, as shown by scientific studies [70] and reported in the CNR (National Research Council) Italian guidelines [71], although there is a reduction of the flexural strength, but fracture tests are necessary to measure actually the toughness and verify its real improvement. However, hemp fibers, due to their deformability (elastic modulus between 23.9 and 90 GPa [34]), cannot give a high toughness to oppose the crack opening like steel fiber-reinforced composites; therefore, a decrease in the load is observed before an efficient crack bridging of hemp fibers occurs.

**Figure 9:**
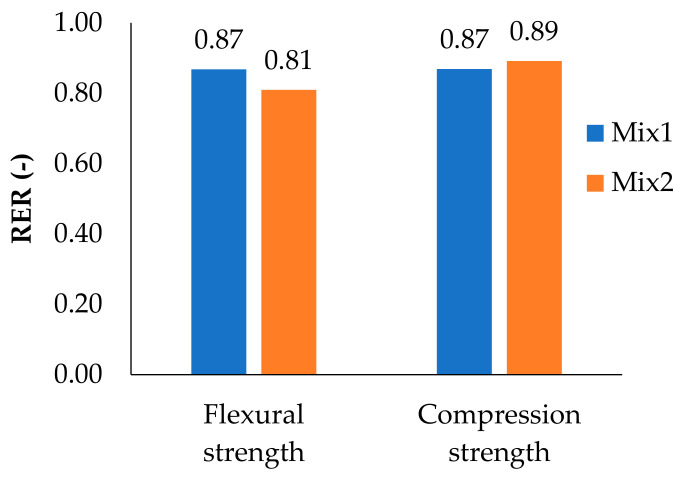
Effect of hemp fibers (RER) on flexural strength and compression strength for NC specimens.

In Table 4 and Table 5, the results of flexural strength and compression strength of NC specimens according to the type of mixture are reported, respectively. In addition, the reinforcement efficient ratio (RER) is calculated as the ratio between the strength of the FR specimens over the respective REF specimens (Figure 9) in order to analyze the influence of the hemp fibers on the mechanical properties.

The results show that the mean value of the compressive strength of each mixture is approximately double compared to the respective mean value of the flexural strength.

Regarding the results in terms of strength, the comparison between the two types of the mixture, both for REF specimens and for FR specimens, shows that values of Mix 1 are on average higher by about 60% compared to Mix 2. In particular, the amount of water introduced into Mix 2, in order to facilitate the manual mixing procedure with hemp fibers, has penalized the binder, leading to the worsening of mechanical strength. As regards the scattering of the result, Mix 1 has lower COVs (6–10%) than Mix 2 (up to 21%).

The different results obtained for the various mixtures highlight the aspect of the uncertainties of the composite properties due to the hand-making influence, both for reinforced and not reinforced mixture.

**Figure 10:**
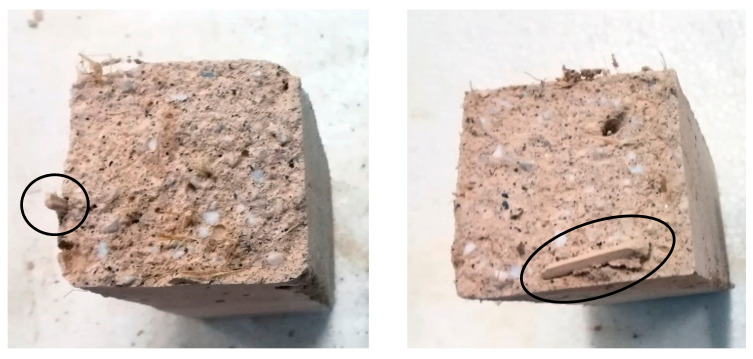
Examples of agglomeration of hemp fibers in tested specimens.

From Figure 9, it is worth noting that the presence of hemp fibers does not lead to an improvement of the mechanical behavior of the mixtures (RER < 1). The ineffectiveness of hemp fibers is mainly due to their distribution in the mixture. Indeed, during the production of the mixtures, it is difficult to achieve a homogeneous distribution of the fibers, and an agglomeration of fibers has been often observed in the broken specimens (Figure 10) [72]. This has favored the creation of discontinuities in the material, which has increased the porosity and locally weakened the specimens, generally leading to a worse mechanical response [73].

### 4.2. Results of Mechanical Tests for C Specimens

Similarly to what has already been described for the NC (not-conditioned) specimens, in this section, the results for the C (conditioned) specimens of Mix 1, both for reference specimens (Mix1_REF) and reinforced specimens (Mix1_FR), and of Mix 2, only for FR specimens (Mix2_FR), are summarized. Detailed results of each specimen are reported in Appendix A. In particular, Table 6 and Table 7 show the results of saline and moisture exposure on the flexural strength and the compression strength, respectively. Besides, in this section, the values of mechanical properties with the respective COVs are reported according to the mixture. Before discussing the results of the specific cases, it is worth noting that the tests on the C specimens provide a greater scattering with a wider range of variability, especially for the compression tested specimens, giving lower reliability of the mean values of the properties. Furthermore, it should be noted that the poor copiousness of the sample conditioned at 3000 h both in saline and moisture environments for Mix 1 leads to less meaningful data, making the interpretation of the results more difficult.

Regarding the effect of hemp reinforcement on C specimens of Mix 1, the presence of hemp fibers, in general, does not lead to alteration of the flexural strength (RER close to unity); however, an unstable behavior occurs for the compression strength. In fact, for the saline conditioning, there is an improvement in these mechanical properties, except for the compression strength at 1000 h of exposure, which has no variation (RER = 1.00). On the other hand, for moisture conditioning, there is a significant reduction in compressive strength (up to 40%), but these results also show the greatest degree of dispersion (COVs up to 32%).

**Figure 11:**
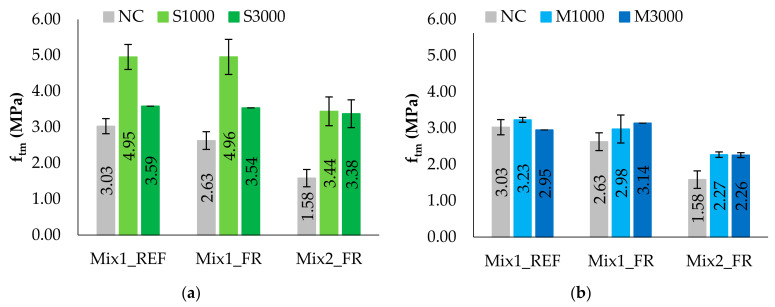
Flexural strength of NC and C specimens: (**a**) saline environment; (**b**) moisture environment.

**Figure 12:**
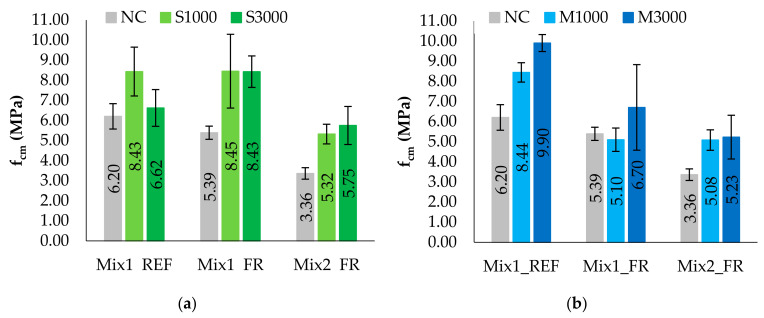
Compression strength of NC and C specimens: (**a**) saline environment, (**b**) moisture environment.

To understand the effects of environmental exposures, the conditioning relationship (CR) is calculated as the ratio between the strength of the C specimens over the respective NC specimens for the mixture with and without hemp reinforcement (Table 8), and the comparison between the results is also reported in Figure 11 and Figure 12, specifically with reference to the mean values of the mixture.

From the following graphics, it is noted that conditioning in the saline environment does not worsen the performance in terms of strength, and, in general, it is verified that the exposure at 1000 h has a greater beneficial effect than the exposure at 3000 h. Indeed, analyzing the behavior of Mix1_REF reveals the increase in flexural strength by 64% and 18%, respectively, for S1000 and S3000; therefore, conditioning with longer times inverts the growth trend. Similar trends of flexural strength occur for Mix 1 and Mix 2, characterized by the presence of hemp fibers. Regarding the compression strength, the values of Mix1_REF increase by 36% and 7%, respectively, for S1000 and S3000, while Mix1_FR is less affected by conditioning times and presents similar results for conditioning procedures at 1000 and 3000 h, but the reliability of the results at 1000 h is limited due to high values of COV up to 22%. Instead, Mix2_FR has a significant increase up to 71% for conditioning at 3000 h.

Similarly, a general increase in terms of strength is also found in moisture conditioning but with more variable trends between exposure times. In fact, REF conditioned specimens of Mix 1 are characterized by a flexural strength—firstly, 7% higher for 1000 h of exposition, and after, 2% lower for 3000 h of exposition compared to the NC state, while for FR specimens of the same mixture, an increase of flexural strength of 13% and 19%, respectively, for M1000 and M3000 occurs. Therefore, the effect of conditioning in a moisture environment on the flexural strength of Mix 1 is less evident than conditioning in a saline environment. Furthermore, Mix2_FR is less affected by conditioning times of moisture exposure, showing an increase of 43% in flexural strength compared to the NC state. Regarding the compression tests, the compression strength is more influenced by moisture conditioning than flexural strength, showing an increase of 36% and 60% in the reference mixture for M1000 and M3000, respectively. Similarly, the Mix2_FR shows a significant increase in the compressive strength, equal to 51% and 56%, respectively, for the times of 1000 h and 3000 h. Furthermore, Mix1_FR shows an inverted trend with a 6% decrease and a 24% increase, respectively, for M1000 and M3000 but with high values of COVs (up to 32%).

In general, comparing the two types of conditioning, exposure in the saline environment leads to higher values of mechanical properties compared to exposure in moisture environment, and Mix2_FR is mainly affected by environmental conditions, showing higher variations in mechanical properties.

From the above description of the results, the variability and unpredictability of the mechanical behavior are evident. This aspect certainly depends on the difficulty of mixing the fibers with homogeneous distribution and on the hand-making procedure, which has an important effect when the mortar has a reaction with the water of the environmental conditioning. Indeed, the reason for the improvement of the strength after both types of exposure conditions can be found in the nature of the hydraulic lime-based mortar used for the experimentation, which has the property of setting and hardening underwater. The mechanical properties of this type of mortar are improved when curing in moisture conditions because a higher value of relative humidity favors a higher hydration degree [74,75]. Therefore, the immersion of the specimens in water allows additional hydration of the binder that does not react during the mixing. The amount of the non-reacted binder depends also on the hand-making procedure both for the normal and fiber-reinforced mortar. In the latter case, the improvement of the strength following the water immersion is higher due to the higher porosity of the fiber-reinforced specimens, as better discussed in the next section, which allows a wider penetration of the water. In fact, the strength of Mix1_FR is lower than those of Mix1_REF in normal exposures, but after the conditioning, the strengths are quite equal for flexure and higher for compression. Moreover, the salt does not deteriorate the performance of the material both because the mortar based on natural aggregates has a weak affinity with the salt and because the high ductility of the pore walls created by the presence of hemp fibers in the mortar has allowed accommodating expansive salt crystallization pressures, as shown by microscopic analysis from previous studies [76].

### 4.3. Results of Thermal Tests

The measured thickness of each panel is reported in Table 9. Herein, the results of thermal conductibility are also reported. Notably, the operative temperature of the proposed samples—considering typical ranges for the buildings—can generally vary between −10 °C and 50 °C in most climatic zones. In such range, λ can undergo significant variations. Therefore, this property has been measured at three temperature levels, i.e., 10 °C, 20 °C, and 50 °C. In this regard, a temperature difference (ΔT) of 6 °C between the hot plate and cold ones is set in all cases because higher values affect the convergence of the measurement procedure, given the quite high ***λ*** values under investigation (see Equation (3)), as shown by tests conducted with higher ΔT. On the other hand, lower values would reduce the results’ accuracy. For each specimen and each temperature, the test has lasted about 4 h.

The first conclusion starting from Table 9 is the variability of conductivity with the operative temperature. However, the hemp fibers seem to have more stable behavior compared with the reference specimen. Moreover, the obtained values unveil that the hemp integration inside the mortar implies a benefit as concerns building energy performance. Indeed, there is a significant λ reduction, around 10% for all temperature levels. These values are not comparable with those available in the scientific literature, but considering the adopted weight of hemp fibers in the mixture and the fabrication procedure that does not assure a homogenous distribution of the fibers, these give encouraging indications for further optimizations. First of all, the low weight percentage of hemp fibers is not more than 0.2% because higher integration could compromise the mechanical performance and also the workability during the mixing process; however, a good efficiency has been obtained due to the mortar and fibers interaction at the interface, as discussed in the following section.

Presently, it is not possible to estimate what could happen with the increase of hemp weight, also considering fibers orientation in the mixture. Surely, the thermal behavior improves, mainly if shorter fibers are used because the interface would increase and, conversely, the mechanical behavior could get worse. The thermal conductivity of porous materials as hemp is governed by the voids that occur from the packing of fibers. Short fibers are more difficult to align and pack densely [77], and for this reason, when a mixture is created, the number of voids increases; meanwhile, the density and the thermal conductivity should decrease.

Considering the stationary condition for a building during the winter, with a set-point temperature of 20 °C for the heating system, the achieved value of thermal conductivity allows increasing insulation property by means of application of a layer of proposed materials. The equivalent value of the thermal resistance depends on the adopted thickness; considering 50 mm, it is 0.11 (m^2^·K)/W. Obviously, the type of product under investigation cannot be understood as a substitute for an insulating material that has very different characteristics and functions. However, it can contribute to increasing the thermal resistance of opaque envelope, and it could be a suitable solution for refurbishment or restoration intervention on vertical opaque walls without affecting architectural features or details of historic buildings. For instance, when the internal side of walls does not have an architectural interest, the application of an insulating plaster on the inner face, with an opportune vapor barrier, is a compatible solution.

## 5. Discussion of the Experimental Results

In the analysis of the experimental results, it must be considered that the thermo-physical as well as the mechanical properties of vegetable fiber and mortar composites not only depend on matrix and fibers, but they are deeply affected by their interaction at the interface. Indeed, the density of the fibers is near 1500 kg/m^3^, and they absorb water nearly 2.5 times their weight [78]. The density is near the value of the selected mortar, and thus, the reduction of thermal conductivity and compressive strength cannot be attributed to a substantial modification of this value but to the increased porosity. Therefore, the weight of the tested specimens is measured both under dry conditions and wet conditions after the extraction of the specimens from the tanks. The bulk density and wet density are reported, considering the volume of the prismatic specimen 160 mm × 40 mm × 40 mm. Apparent porosity and water absorption are also calculated (Table 10).

Bulk density is described as a property related to the internal structure of the material; also known as apparent density, it determines many properties (i.e., thermal conductivity, strength, weight), and it depends mainly on the materials used but also on the density of the compacted mixture. The values reported in Table 10 show that the inclusion of 0.2% of hemp fibers gives a negligible reduction of the mortar density; therefore, the reduction in the mechanical strengths by approximately 10–15% and the reduction in the thermal conductivity by 10% are due to the formation of discontinuity surfaces between the matrix and the fiber, which entrap air during the manual mixing process [78]. This aspect is also confirmed by the value of apparent porosity, reported in Table 10, that is, an increase of approximately 20% for the mixture with hemp fibers.

Comparing the mechanical results of the two mixtures prepared, the worsening of the mechanical strengths of Mix 2 is due to the additional water that has been included to facilitate the manual mixing process. It is a long-known fact that increasing the water-binder ratio increases the porosity of the mixture, which, in turn, decreases the compressive and the tensile strengths [79,80]. However, the higher values of apparent porosity of Mix 2 and, consequently, the higher capacity of water absorption probably lead to an additional hardening of the mixture during the environmental treatment in water; therefore, an improvement in CR coefficient for Mix 2 (Table 8) is observed. Previous research underlined that hemp absorbs most of the water present in the matrix, causing superficial deposition and scarce adhesion of the lime to the hemp; thus, the hardening process is also delayed [81]; therefore, the immersion in water for the environmental conditioning could have reactivated the hardening process and enhanced the mechanical performance.

Furthermore, the plaster investigated is a natural hydraulic lime-based mortar, which is a very porous material that guarantees high vapor diffusion and breathability to the substrate. Previous studies have shown that the water/binder ratio is the most important parameter in controlling the porosity, also influencing the distribution of the pores [82,83]; therefore, porosity, as well as density and water absorption, is significantly affected by the mortar preparation. Surely, it can be established that an increase in porosity reduces the strength of a mortar; however, other authors have claimed that this is only true when the binder is in excess and that, when the binder is not in excess, an increase in porosity enhances carbonation, thus enhancing strength [84].

The experimental results confirm that the mechanical and thermal characteristics of the composite depend not only on the components but also on their interaction. A key parameter to predict or to control the final thermal behavior could be the dispersion of the fibers, which is influenced by the fiber length and dosage and the fiber-matrix bond. As also reported in the literature [85], several processes contribute to the interaction at the fiber-matrix interface, such as fiber mineralization, fiber degradation, and fiber volume change due to water absorption. Considering the morphological structure of hemp fibers by scanning electron microscopy, as reported in several papers [86], the untreated fibers (as in the present study) are rough because these have a lot of compounds at the surface covered with fats, waxes, or polysaccharides. For this reason, when the fibers are mixed into the selected mortar, the porosity of the composite increases, as well as the air gets entrapped during mixing.

The aforementioned considerations highlight the complexity in the evaluation of the effects that the internal structure of the material has on its performance; therefore, a further experimental campaign is necessary to investigate more mixtures, i.e., with different water/binder ratio, also with microscopic analysis in order to achieve the optimization between physical and mechanical properties.

However, some preliminary conclusions can be found considering comparable studies. For instance, Hamzaoui et al. [87] found that the addition of small volume content of hemp fibers (<10%) increased linearly the porosity content in mortar. The rate of porosity largely decreases if wet hemp is used. This phenomenon causes a linear reduction of both stiffness and compressive strength of mortar and probably also a linear reduction of thermal conductivity. Instead, Benmansour et al. [88], considering a mortar with date palm fibers, found that the fiber concentration ratio in the range of 5–15% satisfied both the thermal and mechanical requirements of the composite. In particular, when the weight concentration of fiber is higher than 15%, the size of the fibers on the thermal conductivity of the composite is negligible, but the compressive strength significantly decreases.

## 6. Conclusions

In this article, the experimental results of mechanical and thermal tests on the hemp-lime mortar in normal and conditioned conditions are reported in order to understand the performance of this innovative material increasingly used due to its high environmental benefits. The experimental campaign is a preliminary one limited to one percent of fibers in two mixtures, but some interesting conclusions are proposed in the following:

1. The mixture with a low percentage of fiber (0.2%) assures good workability in the hand application of the mortar as a plaster on a wide surface of a wall.
2. The hand manufacturing procedure of mortars can influence the mechanical properties of the plaster, giving a scattering that simulates the conditions of in situ applications. This variability occurs for specimens with and without fibers.
3. The hemp fibers with a low percentage of 0.2% reduce the strong performance of the mortars (approximately 10–15%) due to the increase in the porosity of the material (approximately 20%) in the process of fibers mixing. This effect is further amplified by the local irregularities in the mixture created by the agglomeration of the fibers.
4. The hemp fibers can improve the cohesive effect in the failure mechanism in tension due to the bridging effect in the cracks evidenced after the failure in flexure.
5. The proposed aging protocols (saline environment and moisture environment) in most cases have not evidenced degradation of the mechanical properties of the material. This result is related to the nature of the binder and the way of conditioning the specimens. In fact, the lime used is a hydraulic binder that can set and harden if immersed in water. The positive effect of conditioning is the same for the case with and without fibers.
6. The thermal conductivity decreases by around 10–11% compared with the same specimen without fibers, allowing an interesting thermal resistance of 0.11 (m^2^·K)/W, considering the application of 50 mm thickness. The insulation property is influenced by the production method and by the not homogeneous distribution of the fiber, which also increases the porosity of the material.

As a final word, nevertheless, the scattering of the results due to the difficult workability of the mixture with the fibers, it is clear that hemp percentage of just 0.2% by weight achieves a reduction of 10% in the thermal conductivity and compressive strength, without a significant reduction in the mechanical properties and degradation under environmental conditioning. Therefore, a correct mixing and manufacturing process of the product can give a material an interesting perspective for thermal benefits and sufficient mechanical characteristics.

However, further experimental tests are necessary to optimize the mixtures and develop a relationship between the thermal and mechanical properties and the fiber weight or length, considering also the elastic properties and the toughness in the fracture process, which are necessary for a full characterization of the composite material.

## Figures and Tables

**Table 1:** The number of specimens tested according to the mixture casted.

Type of Test	Mixture	N° REF Specimens	N° FR Specimens
Mechanical	Mix 1	10	10
	Mix 2	4	14
Thermal	Mix 1	2	2

N°: number; REF: reference; FR: fiber-reinforced.

**Table 2:** Summary of mechanical tests according to the type of mixture, the presence of hemp fibers, and the type of environmental conditioning.

Mixture	REF Specimens	FR Specimens	Conditioning
Mix 1	I1, I2, I3, I4	IFR1, IFR2, IFR3, IFR4	NC
	I5, I6	IFR5, IFR6	S1000
	I7	IFR7	S3000
	I8, I9	IFR8, IFR9	M1000
	I10	IFR10	M3000
Mix 2	I11, I12, I13, I14	IFR11, IFR12, IFR13, IFR14	NC
		IFR1, IFR2	S1000
		IFR3, IFR4, IFR5	S3000
		IFR9, IFR10	M1000
		IFR6, IFR7, IFR8	M3000

**Table 3:** Technical specification of GHP 456 Titan.

Parameter	Specification
Thermal conductivity range	0.003 to 2 W/(m·K)
Minimum measurable thermal resistance	0.02 (m^2^·K)/W
Accuracy	2%
Reproducibility	<1%

**Table 4:** Results of flexural strength of NC specimens according to the type of mixture.

Mixture	REF Specimens		FR Specimens		RER
	f_tm_ (MPa)	COV_t_	f_tm_ (MPa)	COV_t_	
Mix 1	3.03	7%	2.63	9%	0.87
Mix 2	1.96	10%	1.58	15%	0.81

**Table 5:** Results of compression strength of NC specimens according to the type of mixture.

Mixture	REF Specimens		FR Specimens		RER
	f_cm_ (MPa)	COV_c_	f_cm_ (MPa)	COV_c_	
Mix 1	6.20	10%	5.39	6%	0.87
Mix 2	3.77	21%	3.36	9%	0.89

**Table 6:** Results of flexural strength of C specimens subjected to saline and moisture conditioning.

Mixture	Exposure	REF Specimens		FR Specimens		RER
		f_tm_ (MPa)	COV_t_	f_tm_ (MPa)	COV_t_	
Mix 1	S1000	4.95	7%	4.96	10%	1.00
	S3000	3.59	-	3.54	-	0.99
	M1000	3.23	2%	2.98	13%	0.92
	M3000	2.95	-	3.14	-	1.06
Mix 2	S1000	-	-	3.44	12%	-
	S3000	-	-	3.38	11%	-
	M1000	-	-	2.27	3%	-
	M3000	-	-	2.26	3%	-

**Table 7:** Results of compression strength of C specimens subjected to saline and moisture conditioning.

Mixture	Exposure	REF Specimens		FR Specimens		RER
		f_cm_ (MPa)	COV_c_	f_cm_ (MPa)	COV_c_	
Mix 1	S1000	8.43	14%	8.45	22%	1.00
	S3000	6.62	14%	8.43	9%	1.27
	M1000	8.44	6%	5.10	11%	0.60
	M3000	9.90	4%	6.70	32%	0.68
Mix 2	S1000	-	-	5.32	9%	-
	S3000	-	-	5.75	16%	-
	M1000	-	-	5.08	10%	-
	M3000	-	-	5.23	21%	-

**Table 8:** Effect of environmental conditioning (CR) on flexural strength and compression strength.

Exposure	CR—Flexural Strength			CR—Compression Strength		
	Mix1_REF	Mix1_FR	Mix2_FR	Mix1_REF	Mix1_FR	Mix2_FR
S1000	1.64	1.89	2.17	1.36	1.57	1.58
S3000	1.18	1.35	2.13	1.07	1.56	1.71
M1000	1.07	1.13	1.43	1.36	0.95	1.51
M3000	0.98	1.20	1.43	1.60	1.24	1.56

**Table 9:** Measurement results for thermal conductivity.

Specimen	Panel	Thickness (mm)	λ (W/(m·K)) at −10 °C	λ (W/(m·K)) at 20 °C	λ (W/(m·K)) at 50 °C
1 (no hemp)	panel 1	53.5 ± 0.1	0.49 ± 0.01	0.52 ± 0.01	0.55 ± 0.01
	panel 2	51.1 ± 0.1			
2 (with hemp)	panel 1	52.0 ± 0.1	0.44 ± 0.01	0.46 ± 0.01	0.49 ± 0.01
	panel 2	51.5 ± 0.1			
Percentage difference			−10%	−11%	−11%

**Table 10:** Density measurement.

Type of Mixture		Bulk Density (g/cm^3^)	Wet Density (g/cm^3^)	Apparent Porosity (%)	Water Absorption (%)
Mix 1	no hemp	1.88	2.11	23.0	12.2
	with hemp	1.82	2.10	28.3	15.5
Mix 2	no hemp	1.81	2.12	26.1	14.5
	with hemp	1.76	2.10	33.4	19.0

## Data Availability

Data is contained within the article.

## Appendix A

This section reports the results of mechanical tests depending on the two types of mixture casted in the experimental program. For each specimen, the flexural strength f_t_ is calculated according to Equation (1); the compression strength f_c_ is determined according to Equation (2) on the two fragments (identified by subscripts 1 and 2) resulting from the specimens broken by flexural tests. Table A1 and Table A2 show the experimental results as a function of the environmental exposure and the presence of hemp fibers for Mixture 1 and Mixture 2, respectively.

**Table A1:** Detailed results of flexural and compression tests of specimens of Mixture 1.

Exposure	REF Specimens				FR Specimens			
	Flexural Tests		Compression Tests		Flexural Tests		Compression Tests	
	ID	ft (MPa)	ID	fc (MPa)	ID	ft (MPa)	ID	fc (MPa)
NC	I1	2.77	I1,1	5.58	IFR1	2.65	IFR1,1	4.96
			I1,2	5.11			IFR1,2	5.05
	I2	3.28	I2,1	7.20	IFR2	2.60	IFR2,1	5.42
			I2,2	6.29			IFR2,2	5.24
	I3	2.99	I3,1	6.54	IFR3	2.93	IFR3,1	5.59
			I3,2	6.49			IFR3,2	6.01
	I4	3.07	I4,1	6.30	IFR4	2.33	IFR4,1	5.46
			I4,2	6.13			IFR4,2	5.39
S1000	I5	5.20	I5,1	6.86	IFR5	5.30	IRF5,1	10.94
			I5,2	8.14			IRF5,2	7.03
	I6	4.71	I6,1	9.14	IFR6	4.61	IRF6,1	8.74
			I6,2	9.61			IRF6,2	7.10
S3000	I7	3.59	I7,1	5.98	IFR7	3.54	IRF7,1	7.88
			I7,2	7.27			IRF7,2	8.98
M1000	I8	3.18	I8,1	8.66	IFR8	2.71	IRF8,1	4.80
			I8,2	7.92			IRF8,2	5.67
	I9	3.28	I9,1	9.00	IFR9	3.25	IRF9,1	4.43
			I9,2	8.19			IRF9,2	5.49
M3000	I10	2.95	I10,1	10.2	IFR10	3.14	IRF10,1	5.20
			I10,2	9.60			IRF10,2	8.21

**Table A2:** Detailed results of flexural and compression tests of specimens of Mixture 2.

Exposure	REF Specimens				FR Specimens			
	Flexural Tests		Compression Tests		Flexural Tests		Compression Tests	
	ID	f_t_ (MPa)	ID	f_c_ (MPa)	ID	f_t_ (MPa)	ID	f_c_ (MPa)
NC	I11	2.26	I11,1	5.18	IFR11	1.84	IFR11,1	2.88
			I11,2	4.59			IFR11,2	3.83
	I12	1.87	I12,1	3.13	IFR12	1.27	IFR12,1	3.31
			I12,2	3.29			IFR12,2	3.53
	I13	1.88	I13,1	3.30	IFR13	1.54	IFR13,1	3.42
			I13,2	4.04			IFR13,2	3.11
	I14	1.82	I14,1	3.77	IFR14	1.68	IFR14,1	3.51
			I14,2	2.86			IFR14,2	3.29
S1000	-				IFR1	3.16	IFR1,1	5.74
							IFR1,2	4.66
	-				IFR2	3.72	IFR2,1	5.65
							IFR2,2	5.24
S3000	-				IFR3	3.56	IFR3,1	5.65
							IFR3,2	4.07
	-				IFR4	2.93	IFR4,1	5.6
							IFR4,2	5.93
	-				IFR5	3.63	IFR5,1	6.47
							IFR5,2	6.79
M1000	-				IFR9	2.22	IFR9,1	5.47
							IFR9,2	5.17
	-				IFR10	2.32	IFR10,1	5.35
							IFR10,2	4.34
M3000	-				IFR6	2.30	IFR6,1	6.34
							IFR6,2	5.85
	-				IFR7	2.30	IFR7,1	4.31
							IFR7,2	6.43
	-				IFR8	2.18	IFR8,1	4.27
							IFR8,2	4.18

## References

[1] Joshi S., Drzal L., Mohanty A., Arora S. (2004). Are natural fiber composites environmentally superior to glass fiber reinforced composites?. Compos. Part A Appl. Sci. Manuf..

[2] Naidu A.L., Jagadeesh V., Bahubalendruni M.R. (2017). A review on chemical and physical properties of natural fiber reinforced composites. J. Adv. Res. Eng. Technol..

[3] Fidelis M.E.A., Pereira T.V.C., Gomes O.D.F.M., Silva F.D.A., Filho R.D.T. (2013). The effect of fiber morphology on the tensile strength of natural fibers. J. Mater. Res. Technol..

[4] Iucolano F., Liguori B., Aprea P., Caputo D. (2018). Thermo-mechanical behaviour of hemp fibers-reinforced gypsum plasters. Constr. Build. Mater..

[5] Peijs T. (2002). Composites turn green. Polymers.

[6] Chen C., Yang Y., Yu J., Yu J., Tan H., Sui L., Zhou Y. (2020). Eco-friendly and mechanically reliable alternative to synthetic FRP in externally bonded strengthening of RC beams: Natural FRP. Compos. Struct..

[7] Chen C., Yang Y., Zhou Y., Xue C., Chen X., Wu H., Sui L., Li X. (2020). Comparative analysis of natural fiber reinforced polymer and carbon fiber reinforced polymer in strengthening of reinforced concrete beams. J. Clean. Prod..

[8] Corbière-Nicollier T., Laban B.G., Lundquist L., Leterrier Y., Månson J.-A., Jolliet O. (2001). Life cycle assessment of biofibres replacing glass fibres as reinforcement in plastics. Resour. Conserv. Recycl..

[9] Zhou C., Shi S.Q., Chen Z., Cai L., Smith L. (2018). Comparative environmental life cycle assessment of fiber reinforced cement panel between kenaf and glass fibers. J. Clean. Prod..

[10] Sen T., Reddy H.J. (2014). Efficacy of bio derived jute FRP composite based technique for shear strength retrofitting of reinforced concrete beams and its comparative analysis with carbon and glass FRP shear retrofitting schemes. Sustain. Cities Soc..

[11] European Commission Common Catalogue of Varieties of Agricultural Plant Species. https://op.europa.eu/.

[12] European Commission Delegated Regulation (EU) No. 639/2014 of March 11. https://eur–lex.europa.eu/.

[13] Shahzad A. (2012). Hemp fiber and its composites—A review. J. Compos. Mater..

[14] Cazacu C., Muntean R., Gălățanu T., Taus D. (2016). Hemp Lime Technology.

[15] Campiglia E., Gobbi L., Marucci A., Rapa M., Ruggieri R., Vinci G. (2020). Hemp Seed Production: Environmental Impacts of *Cannabis Sativa L*. Agronomic Practices by Life Cycle Assessment (LCA) and Carbon Footprint Methodologies. Sustainability.

[16] Schumacher A.G.D., Pequito S., Pazour J. (2020). Industrial hemp fiber: A sustainable and economical alternative to cotton. J. Clean. Prod..

[17] Iucolano F., Boccarusso L., Langella A. (2019). Hemp as eco-friendly substitute of glass fibres for gypsum reinforcement: Impact and flexural behaviour. Compos. Part B Eng..

[18] Gaujena B., Agapovs V., Borodinecs A., Strelets K. (2020). Analysis of Thermal Parameters of Hemp Fiber Insulation. Energies.

[19] Pickering K., Efendy M.A., Le T. (2016). A review of recent developments in natural fibre composites and their mechanical performance. Compos. Part A Appl. Sci. Manuf..

[20] Yan L., Kasal B., Huang L. (2016). A review of recent research on the use of cellulosic fibres, their fibre fabric reinforced cementitious, geo-polymer and polymer composites in civil engineering. Compos. Part B Eng..

[21] Shahzad A. (2013). A Study in Physical and Mechanical Properties of Hemp Fibres. Adv. Mater. Sci. Eng..

[22] Ingrao C., Giudice A.L., Bacenetti J., Tricase C., Dotelli G., Fiala M., Siracusa V., Mbohwa C. (2015). Energy and environmental assessment of industrial hemp for building applications: A review. Renew. Sustain. Energy Rev..

[23] Shea A., Lawrence M., Walker P. (2012). Hygrothermal performance of an experimental hemp–lime building. Constr. Build. Mater..

[24] Le A.D.T., Maalouf C., Mai T., Wurtz E., Collet F. (2010). Transient hygrothermal behaviour of a hemp concrete building envelope. Energy Build..

[25] Summerscales J., Dissanayake N.P., Virk A.S., Hall W. (2010). A review of bast fibres and their composites. Part 1—Fibres as reinforcements. Compos. Part A Appl. Sci. Manuf..

[26] Summerscales J., Dissanayake N., Virk A., Hall W. (2010). A review of bast fibres and their composites. Part 2—Composites. Compos. Part A Appl. Sci. Manuf..

[27] Sassoni E., Manzi S., Motori A., Montecchi M., Canti M. (2014). Novel sustainable hemp-based composites for application in the building industry: Physical, thermal and mechanical characterization. Energy Build..

[28] Li Z., Wang X., Wang L. (2006). Properties of hemp fibre reinforced concrete composites. Compos. Part A Appl. Sci. Manuf..

[29] Benfratello S., Capitano C., Peri G., Rizzo G., Scaccianoce G., Sorrentino G. (2013). Thermal and structural properties of a hemp–lime biocomposite. Constr. Build. Mater..

[30] Zampori L., Dotelli G., Vernelli V. (2013). Life Cycle Assessment of Hemp Cultivation and Use of Hemp-Based Thermal Insulator Materials in Buildings. Environ. Sci. Technol..

[31] Kymäläinen H.-R., Sjöberg A.-M. (2008). Flax and hemp fibres as raw materials for thermal insulations. Build. Environ..

[32] Fassi A., Maina L. (2009). Guida all’uso dei materiali naturali. L’isolamento Ecoefficiente.

[33] Bevan R., Woolley T. (2008). Hemp Lime Construction: A Guide to Building with Hemp Lime Composites.

[34] Daly P., Ronchetti P., Woolley T. (2021). Hemp Lime Bio–Composite as a Building Material Irish Construction.

[35] Florentin Y., Pearlmutter D., Givoni B., Gal E. (2017). A life-cycle energy and carbon analysis of hemp-lime bio-composite building materials. Energy Build..

[36] Latif E., Lawrence R., Shea A., Walker P. (2015). Moisture buffer potential of experimental wall assemblies incorporating formulated hemp-lime. Build. Environ..

[37] Degrave-Lemeurs M., Glé P., De Menibus A.H. (2018). Acoustical properties of hemp concretes for buildings thermal insulation: Application to clay and lime binders. Constr. Build. Mater..

[38] Garkhail S.K., Heijenrath R.W.H., Peijs T. (2000). Mechanical Properties of Natural-Fibre-Mat- Reinforced Thermoplastics based on Flax Fibres and Polypropylene. Appl. Compos. Mater..

[39] Brzyski P., Grudzińska M., Majerek D. (2019). Analysis of the Occurrence of Thermal Bridges in Several Variants of Connections of the Wall and the Ground Floor in Construction Technology with the Use of a Hemp-lime Composite. Materials.

[40] Barclay M., Holcroft N., Shea A. (2014). Methods to determine whole building hygrothermal performance of hemp–lime buildings. Build. Environ..

[41] E Walker R., Pavia S. (2014). Moisture transfer and thermal properties of hemp–lime concretes. Constr. Build. Mater..

[42] Brzyski P., Łagód G. (2018). Physical and mechanical properties of composites based on hemp shives and lime. E3S Web Conf..

[43] Kremensas A., KairytĖ A., Vaitkus S., Vėjelis S., Balčiūnas G. (2019). Mechanical Performance of Biodegradable Thermoplastic Polymer-Based Biocomposite Boards from Hemp Shivs and Corn Starch for the Building Industry. Materials.

[44] Diquélou Y., Gourlay E., Arnaud L., Kurek B. (2016). Influence of binder characteristics on the setting and hardening of hemp lightweight concrete. Constr. Build. Mater..

[45] Elfordy S., Lucas F., Tancret F., Scudeller Y., Goudet L. (2008). Mechanical and thermal properties of lime and hemp concrete (“hempcrete”) manufactured by a projection process. Constr. Build. Mater..

[46] Nguyen T.T., Picandet V., Carre P., Lecompte T., Amziane S., Baley C. (2010). Effect of compaction on mechanical and thermal properties of hemp concrete. Eur. J. Environ. Civ. Eng..

[47] Nguyen T.-T., Picandet V., Amziane S., Baley C. (2009). Influence of compactness and hemp hurd characteristics on the mechanical properties of lime and hemp concrete. Eur. J. Environ. Civ. Eng..

[48] Arnaud L., Gourlay E. (2012). Experimental study of parameters influencing mechanical properties of hemp concretes. Constr. Build. Mater..

[49] Le A., Gacoin A., Li A., Mai T., Rebay M., Delmas Y. (2014). Experimental investigation on the mechanical performance of starch–hemp composite materials. Constr. Build. Mater..

[50] Çomak B., Bideci A., Bideci Ö.S. (2018). Effects of hemp fibers on characteristics of cement based mortar. Constr. Build. Mater..

[51] Abdellatef Y., Khan M.A., Khan A., Alam M.I., Kavgic M. (2020). Mechanical, Thermal, and Moisture Buffering Properties of Novel Insulating Hemp-Lime Composite Building Materials. Materials.

[52] Filho R.D.T., Ghavami K., England G.L., Scrivener K. (2003). Development of vegetable fibre–mortar composites of improved durability. Cem. Concr. Compos..

[53] Asprone D., Durante M., Prota A., Manfredi G. (2011). Potential of structural pozzolanic matrix–hemp fiber grid composites. Constr. Build. Mater..

[54] Brzyski P., Barnat-Hunek D., Suchorab Z., Łagód G. (2017). Composite Materials Based on Hemp and Flax for Low-Energy Buildings. Materials.

[55] Curto D., Guercio A., Franzitta V. (2020). Investigation on a Bio-Composite Material as Acoustic Absorber and Thermal Insulation. Energies.

[56] Pochwała S., Makiola D., Anweiler S., Böhm M. (2020). The Heat Conductivity Properties of Hemp–Lime Composite Material Used in Single-Family Buildings. Materials.

[57] Glé P., Gourdon E., Arnaud L. (2011). Acoustical properties of materials made of vegetable particles with several scales of porosity. Appl. Acoust..

[58] Collet F., Pretot S. (2014). Thermal conductivity of hemp concretes: Variation with formulation, density and water content. Constr. Build. Mater..

[59] Collet F., Chamoin J., Pretot S., Lanos C. (2013). Comparison of the hygric behaviour of three hemp concretes. Energy Build..

[60] Posani M., Veiga M.D.R., De Freitas V.P. (2019). Towards Resilience and Sustainability for Historic Buildings: A Review of Envelope Retrofit Possibilities and a Discussion on Hygric Compatibility of Thermal Insulations. Int. J. Arch. Heritage.

[61] Bruijn P.S.-D., Donarelli A., Balksten K. (2019). Full-scale Studies of Improving Energy Performance by Renovating Historic Swedish Timber Buildings with Hemp-lime. Appl. Sci..

[62] Agliata R., Marino A., Mollo L., Pariso P. (2020). Historic Building Energy Audit and Retrofit Simulation with Hemp-Lime Plaster—A Case Study. Sustainability.

[63] European Standards UNI EN 459–1 (2001). Building Lime–Definitions, Specifications and Conformity Criteria.

[64] European Standards UNI EN 998–2 (2001). Specification for Mortar for Masonry—Part 2: Masonry Mortar.

[65] Ascione F., De Masi R.F., De Rossi F., Ruggiero S., Vanoli G.P. (2016). MATRIX, a multi activity test-room for evaluating the energy performances of ‘building/HVAC’ systems in Mediterranean climate: Experimental set-up and CFD/BPS numerical modeling. Energy Build..

[66] European Standards UNI EN 1015–11 (2007). Methods of Test for Mortar for Masonry—Determination of Flexural and Compressive Strength of Hardened Mortar.

[67] ASTM D1141–98 (2008). Standard Practice for the Preparation of Substitute Ocean Water.

[68] Thermal Conductivity Sensors. https://www.netzsch-thermal-analysis.com/en/.

[69] ISO 8302 (1991). Thermal Insulation. Determination of Steady State Thermal Resistance and Related Properties. Guarded Hot Plate Apparatus.

[70] Li V.C., Maalej M. (1996). Toughening in cement based composites. Part I: Cement, mortar, and concrete. Cem. Concr. Compos..

[71] CNR–DT 204/2006 (2006). Guide for the Design and Construction of Fiber–Reinforced Concrete Structures, Design Recommendation.

[72] Kesikidou F., Stefanidou M. (2019). Natural fiber-reinforced mortars. J. Build. Eng..

[73] Hamzaoui R., Guessasma S., Abahric K. Mechanical performance of mortars modified with hemp fibres, shives and milled fly ashes. Proceedings of the 2nd International RILEM/COST Conference on Early Age Cracking and Serviceability in Cement–based Materials and Structures—EAC2.

[74] Arizzi A., Martinez-Huerga G., Sebastian-Pardo E., Cultrone G. (2015). Estudio mineralógico, textural y físico-mecánico de morteros de cal hidráulica curados en distintas condiciones de humedad relativa. Mater. Constr..

[75] Grilo J., Faria P., Veiga R., Silva A.S., Silva V., Velosa A. (2014). New natural hydraulic lime mortars—Physical and microstructural properties in different curing conditions. Constr. Build. Mater..

[76] Walker R., Pavia S., Mitchell R. (2014). Mechanical properties and durability of hemp-lime concretes. Constr. Build. Mater..

[77] Almaadeed M.A., Kahraman R., Khanam P.N., Madi N. (2012). Date palm wood flour/glass fibre reinforced hybrid composites of recycled polypropylene: Mechanical and thermal properties. Mater. Des..

[78] Poletanovic B., Dragas J., Ignjatovic I., Komljenovic M., Merta I. (2020). Physical and mechanical properties of hemp fibre reinforced alkali-activated fly ash and fly ash/slag mortars. Constr. Build. Mater..

[79] Birchall J.D., Howard A.J., A Kendall K. (1981). Flexural strength and porosity of cements. Nat. Cell Biol..

[80] Yudenfreund M., Odler I., Brunauer S. (1972). Hardened portland cement pastes of low porosity I. Materials and experimental methods. Cem. Concr. Res..

[81] Arizzi A., Cultrone G., Brummer M., Viles H. (2015). A chemical, morphological and mineralogical study on the interaction between hemp hurds and aerial and natural hydraulic lime particles: Implications for mortar manufacturing. Constr. Build. Mater..

[82] Mosquera M.J., Benítez D., Perry S.H. (2002). Pore structure in mortars applied on restoration: Effect on properties relevant to decay of granite buildings. Cem. Concr. Res..

[83] Papayianni I., Stefanidou M. (2006). Strength–porosity relationships in lime–pozzolan mortars. Constr. Build. Mater..

[84] Lanas J., Bernal J.P., Bello M., Galindo J.A. (2004). Mechanical properties of natural hydraulic lime-based mortars. Cem. Concr. Res..

[85] Candamano S., Crea F., Coppola L., De Luca P., Coffetti D. (2020). Influence of acrylic latex and pre-treated hemp fibers on cement based mortar properties. Constr. Build. Mater..

[86] Sair S., Oushabi A., Kammouni A., Tanane O., Abboud Y., Hassani F.O., Laachachi A., El Bouari A. (2017). Effect of surface modification on morphological, mechanical and thermal conductivity of hemp fiber: Characterization of the interface of hemp –Polyurethane composite. Case Stud. Therm. Eng..

[87] Hamzaoui R., Guessasma S., Mecheri B., Eshtiaghi A.M., Bennabi A. (2014). Microstructure and mechanical performance of modified mortar using hemp fibres and carbon nanotubes. Mater. Des..

[88] Benmansour N., Agoudjil B., Gherabli A., Kareche A., Boudenne A. (2014). Thermal and mechanical performance of natural mortar reinforced with date palm fibers for use as insulating materials in building. Energy Build..

